# Enhanced surface nanoanalytics of transient biomolecular processes

**DOI:** 10.1126/sciadv.abq3151

**Published:** 2023-01-13

**Authors:** Alyssa Miller, Sean Chia, Zenon Toprakcioglu, Tuuli Hakala, Roman Schmid, Yaduo Feng, Tadas Kartanas, Ayaka Kamada, Michele Vendruscolo, Francesco Simone Ruggeri, Tuomas P. J. Knowles

**Affiliations:** ^1^ Yusuf Hamied Department of Chemistry, University of Cambridge, Lensfield Road, Cambridge CB2 1EW, UK.; ^2^ Laboratory of Organic Chemistry, Wageningen University and Research, Stippeneng 4, Wageningen, 6703 WE, Netherlands.; ^3^ Physical Chemistry and Soft Matter, Wageningen University and Research, Stippeneng 4, Wageningen, 6703 WE, Netherlands.; ^4^ Cavendish Laboratory, University of Cambridge, Cambridge CB3 0HE, UK.

## Abstract

Fundamental knowledge of the physical and chemical properties of biomolecules is key to understanding molecular processes in health and disease. Bulk and single-molecule analytical methods provide rich information about biomolecules but often require high concentrations and sample preparation away from physiologically relevant conditions. Here, we present the development and application of a lab-on-a-chip spray approach that combines rapid sample preparation, mixing, and deposition to integrate with a range of nanoanalytical methods in chemistry and biology, providing enhanced spectroscopic sensitivity and single-molecule spatial resolution. We demonstrate that this method enables multidimensional study of heterogeneous biomolecular systems over multiple length scales by nanoscopy and vibrational spectroscopy. We then illustrate the capabilities of this platform by capturing and analyzing the structural conformations of transient oligomeric species formed at the early stages of the self-assembly of α-synuclein, which are associated with the onset of Parkinson’s disease.

## Teaser

Maintaining the heterogeneity and structural integrity of monomers and oligomers enables their quantitative study.

## INTRODUCTION

Despite substantial technological advances in the past few decades, the study of the properties of biomolecules under physiologically relevant solution conditions still represents a major challenge in the characterization of biomolecular processes and for material science applications. Classical bulk analytical approaches such as circular dichroism, mass spectrometry, and vibrational and nuclear magnetic resonance spectroscopies enable chemical identification and structural characterization. However, they typically require specific sample pretreatment or high concentrations and specific buffer conditions that exceed the typical ranges found in functional biological systems.

An alternative to these challenges is offered by surface-based techniques, which are powerful tools to study the structure and conformation of biomolecules. They offer chemical identification and characterization, such as Raman and infrared (IR) vibrational spectroscopies in attenuated total reflection (ATR-IR), and visualization, such as atomic force microscopy (AFM) and electron microscopy (EM). These well-established surface-based techniques allow for the determination of structural, chemometric, and single-molecule characterization of biomolecular processes. However, the deposition of a sample on a surface represents an intrinsic limitation of these surface methods because of effects of differential absorption and mass transport phenomena on the surface (*1*, *2*). For a liquid solution on a surface, the differential interaction and absorption of biomolecules with the surface limit the ability to perform analysis on heterogeneous samples, as this selective adsorption results in only a partial representation of the sample composition in bulk solution. This is particularly relevant for single-molecule microscopy and surface-based spectroscopies, because species with potential biological relevance may not be observed, therefore confounding the results (*3*).

Dry conditions also enable analytical measurements that are difficult to conduct in an aqueous environment, such as in the case of nanoscopy and vibrational spectroscopy. However, the removal of the solvent can again introduce artifacts associated with selective adsorption on the surface (*1*, *4*). Furthermore, long incubation times while drying on surfaces can lead to conformational changes, artificial self-assembly, aggregation, and interactions with the surface itself, meaning that the conformation in solution is no longer accurately represented (*5*–*11*). This effect can be observed in bulk spectroscopy measurements, which are frequently performed in air to achieve higher sensitivity. It is generally accepted that the drying process may alter the secondary structure of molecules of interest, especially biomolecules that are extremely sensitive to hydration state (*2*). These combined limitations prevent the robust assessment of biomolecular structures using bulk and single-molecule surface-based methods. Thus, there is an unmet need to overcome the limitations associated with the manual sample preparation for surface-based techniques and allow accurate and reproducible analytical characterization of heterogeneous biomolecular processes.

Here, we report the development of a lab-on-a-chip microfluidic system to overcome some of the challenges associated with surface-based techniques. This method integrates advances in microfluidic spray deposition with lab-on-a-chip capabilities, allowing real-time passive mixing, solution changes, and dilution of the sample before deposition for quantitative nanoscopic, single-molecule, and chemometric analysis (*1*, *12*). We apply this method to study heterogeneous biomolecular systems over multiple length scales, preserving their conformation in physiological-like buffers and concentrations. We demonstrate that this approach enables the study of samples by several techniques, including EM, IR spectroscopy, and AFM. Lab-on-a-chip capabilities allow for on-chip mixing of inorganic, organic, and biological samples for single-step staining and deposition for EM, thus allowing analysis of morphology while accurately representing sample heterogeneity. Furthermore, we exploit the fast evaporation of droplets generated using the microfluidic spray device to achieve a 100-fold improvement in the sensitivity of commercial vibrational spectroscopy instruments and reach single-nanogram protein detection in salt-containing solutions. This procedure enables the preservation and study of the molecular conformations of intrinsically disordered and globular proteins at near-physiological conditions. Finally, we demonstrate that these capabilities offer quantitative information about the conformations of an amyloid-forming protein involved in the onset of Parkinson’s disease and show that we can study the oligomeric species present at the early molecular events of self-assembly, which are generally associated with cellular toxicity.

## RESULTS

### General lab-on-a-chip microfluidic spray deposition

**Fig. 1.**
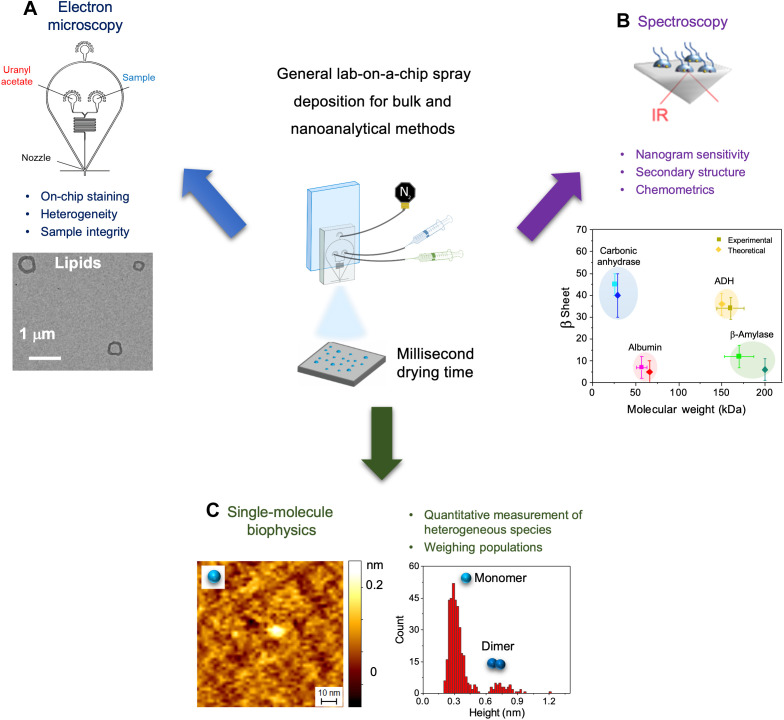
Summary of the applications of the lab-on-a-chip spray deposition method presented in this article. (**A**) In EM, lab-on-a-chip capabilities are exploited to have on-chip mixing of sample with uranyl acetate stain, allowing for a single-step deposition of a range of materials, while being sufficiently gentle to maintain sample integrity. (**B**) In vibrational spectroscopies, microfluidic spray deposition is applied to the secondary structure and chemometric analysis of proteins in salt-containing solution with unprecedented sensitivity. (**C**) In scanning probe microscopy, α-synuclein was deposited via microfluidic spray such that the heterogeneity and relative weights of conformational species are represented as they are in solution, thereby enabling their quantitative assessment.

We developed a method with lab-on-a-chip capabilities for sample deposition on surfaces to improve accuracy and reproducibility of a wide range of microscopic and spectroscopic techniques (Fig. 1). We designed our devices to allow passive two-solution mixing on-chip before being intersected with the gas phase to generate droplets (fig. S1) (*13*). This allowed for on-chip mixing of solutions and immediate deposition onto the surface with no need for the operator to make changes to the initial experimental setup (fig. S2).

Then, we standardized the spray deposition to ensure high levels of reproducibility and accuracy in the amount and area sprayed between different experiments and operators (fig. S3 and note S2). We studied the effect of distance between the device and the surface by varying the distance between 3.5 and 5.5 cm, as this affects the in-flight drying and therefore the droplet size that lands on the surface (notes S3 and S4) (*12*). We investigated the effect of changing flow rate (50 to 500 μl/hour) and gas pressure (0.5 to 4.5 Mbar) to understand their effects on droplet sizes and distributions. This allowed us to determine an optimal, standardized setup, allowing fine-tuning of the deposition and greatly improving reproducibility. To further optimize the spraying capabilities, we verified that most of the sprayed material was distributed in a focused area (>90% in a 1-cm radius area), thus minimizing sample loss and making it particularly well-suited to the handling of valuable biological material. We set up the spray geometry to obtain a small area of spraying, corresponding to the size of the analytical surface in question, such as a 2-mm^2^ prism in the case of Fourier transform IR spectroscopy in ATR (FTIR-ATR). This procedure is demonstrated by the study of the distribution of droplets generated using the spray devices when a solution containing a dye (fluorescein) was sprayed onto a glass coverslip and imaged using fluorescence microscopy. The sprayed droplets exhibited a Gaussian distribution of the fluorescence intensity, with most samples being deposited in a central 5-mm^2^ area and with an approximate total spray radius of 1.5 cm (fig. S3). In all the following experiments, the spraying was optimized to generate a single layer of small droplets on the surface, with diameter in the 3- to 20-μm range, which undergo fast (milliseconds) drying time and so that coalescence of droplets does not occur (note S3) (*1*).

### Lab-on-a-chip deposition for micro- and nanoscopic analysis over multiple length scales

Microscopic and nanoscopic methods such as EM are powerful high-throughput tools used in most chemistry and biology laboratories for investigating biomolecular material and processes (*14*). Moreover, with the recent advent of cryo-EM, EM has become a standard method for the assessment and routine screenings of bio-organic samples before more detailed and higher-resolution experiments (*14*, *15*). However, sample preparation for EM remains one of the most challenging aspects of the technique, particularly when comparison is required with samples analyzed in a vitrified state by cryo-EM. Careful deposition is required to prepare samples, leaving room for possible discrepancies between individual preparations (*16*). This is particularly relevant in the case of negative-stain transmission EM (TEM) experiments, where poor staining of nonconductive biological material and large-stain deposits hinder high-throughput visualization and analysis. As rinsing is required to remove excess sample and stain, the data may not reflect the full sample composition due to differential adsorption effects of heterogeneous biological material.

**Fig. 2.**
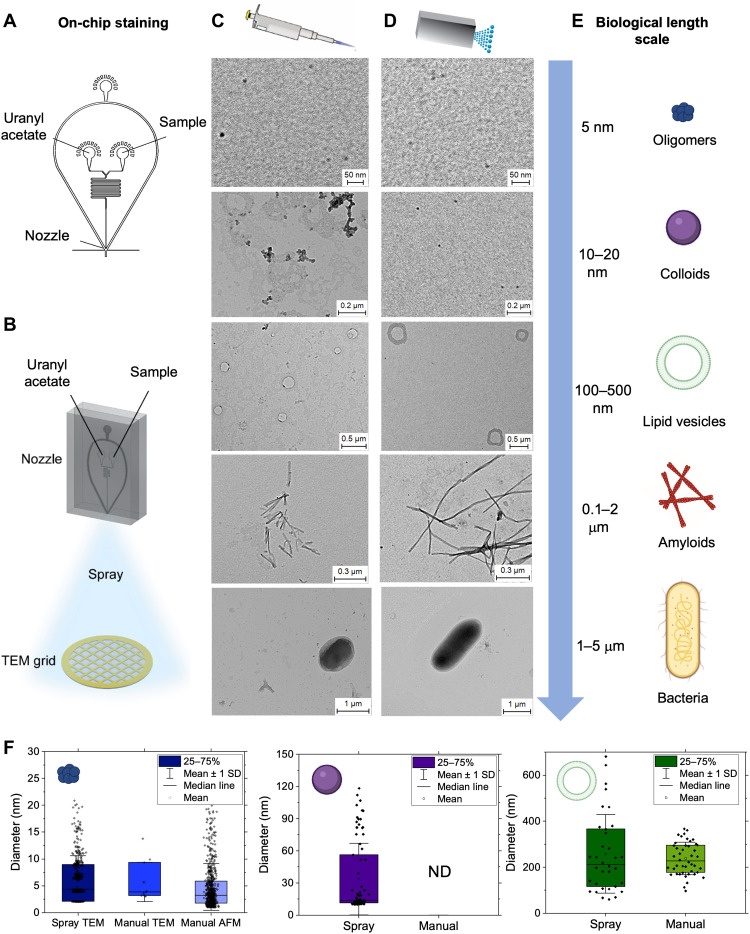
Lab-on-a-chip microfluidic deposition of biological samples over multiple biological length scales for EM. (**A** and **B**) A double-inlet spray nozzle was developed to allow for on-chip staining. One solution channel contained the sample, and the other contained the stain, allowing for a single-step deposition. (**C** to **E**) Comparison of conventional manual deposition (C) and lab-on-a-chip microfluidic spray deposition (D) for organic molecules, biomolecules, and organisms over multiple biological scales (E). (**F**) The diameters of oligomers (left), colloids (center), and lipid vesicles (right) were measured and compared, with a larger range of sizes typically reported for samples prepared via microfluidic spray deposition, indicating that some species present in solution are not represented when deposition involves a rinsing step. Error bars represent the SD of the average measured diameter for each species. ND, not determined.

To overcome these types of limitations of conventional sample preparation for EM, we first demonstrate the application of our microfluidic spray to characterize organic, biological samples and organisms over a wide range of biological length scales (Fig. 2). We exploit the lab-on-a-chip capabilities of our double-inlet spray device to directly stain samples on-chip (Fig. 2A). One channel containing the sample and one channel containing uranyl acetate stain were intersected and mixed on-chip before being sprayed directly onto TEM grids. Thus, we create a single-step sample preparation protocol for EM with a high degree of reproducibility between operators (Fig. 2B). No rinsing step was performed before imaging and analysis.

To prove the capabilities of our approach, we compared TEM measurements of samples from a few nanometers to several micrometers prepared by manual deposition (Fig. 2C) and lab-on-a-chip microfluidic spray (Fig. 2D). Manual deposition involved carefully performed sample deposition and staining steps (see Materials and Methods). We considered the following species in order of length scale (Fig. 2E): amyloidogenic protein oligomers [amyloid-β (Aβ), ~5 nm], colloids formed from the aggregation of a small molecule (bexarotene, ~10 to 20 nm) (*17*), lipid vesicles (phosphatidylcholine and phosphatidylserine, ~100 to 500 nm), Aβ fibrils (~0.1 to 2 μm), and individual bacteria (*Escherichia coli*, ~1 to 5 μm).

In our hands, the manual preparation was not able to successfully prepare all samples. The colloids aggregated during drying, as previously reported (*18*). The manual deposition was also not sufficiently gentle to maintain the integrity of lipid vesicles, which often show deformation effects due to the drying process (Fig. 2C and fig. S5) (*19*, *20*). The lab-on-a-chip spray deposition allowed us to successfully prepare all samples. The single-step spray deposition resulted in samples with minimal excess of uranyl acetate stain deposits on the surface, so a rinsing step was not required (Fig. 2D). This minimizes the effect of differential adsorption on the analysis of heterogeneous biomolecules caused by manual preparations. Thus, our lab-on-a-chip, single-step microfluidic deposition enables us to accurately study the full breadth of populations present in each heterogeneous sample.

Comparing to manual deposition methods, we see a broader size distribution of oligomers and lipid vesicles, indicating that particular species are preserved in spray deposition, which are lost in manual deposition methods (Fig. 2F). Thus, we aimed at quantitatively demonstrating that the sample preparation is improved when deposited via lab-on-a-chip spray, compared to manual deposition. We first performed a single-molecule statistical analysis of the molecules on the surface prepared via the two methods and then compared these results to the dimensions of the molecules in solution as measured by bulk dynamic light scattering (DLS). We considered the case of the oligomeric, colloidal, and lipid samples (Fig. 2F and fig. S4). For all samples deposited via microfluidic spray, the measured size distribution corresponded well to the distributions measured in solution, while manual deposition measurements displayed a narrower size distribution and represented less sample heterogeneity compared to solution measurements (note S5).

**Fig. 3.**
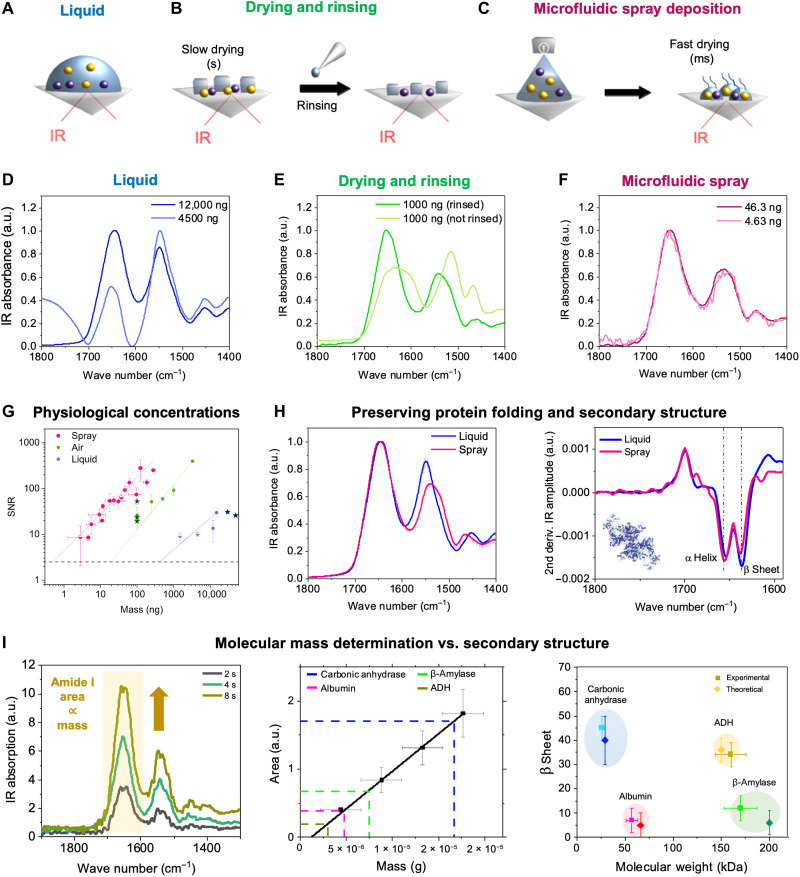
Protein identification by molecular weight and structure at physiological concentrations with nanogram sensitivity. Spectra of protein acquired by (**A**) manual deposition of a macroscopic liquid droplet, (**B**) manual drying and rinsing of a macroscopic liquid droplet, and (**C**) lab-on-a-chip microfluidic spray deposition. Spectra of protein with variable molecular weight acquired at various concentrations in liquid (**D**), air (**E**), and spray (**F**) (figs. S6 to S8). (**G**) Sensitivity in mass (in nanograms) versus signal-to-noise ratio (SNR) of the spectra for the different methods in (A) to (C). The spray deposition enables the detection of nanogram amounts of protein. The SD error on the mass deposited was calculated considering a spray time error of ±1 s (see Materials and Methods). The sensitivity on our measurements (circles) is compared to the sensitivity of state-of-the-art FTIR measurements in literature (darker stars) (*23*–*25*). (**H**) Lab-on-a-chip microfluidic spray preserves molecular conformation as under liquid conditions (left), as demonstrated by the second derivative analysis of the amide I band (right). (**I**) The area of the amide I band is proportional to the amount of protein deposited onto the ATR prism, as shown for a 11.9 μM thyroglobulin solution as a function of three different spray times (left). The fit of the integrated areas is used as calibration curve to determine unknown protein molecular weight (middle and Materials and Methods). The determined molecular weight and protein secondary structure can be used for multiparameter protein identification, as shown in the right panel of protein molecular weight versus secondary structure for several proteins. a.u., arbitrary units, ADH, alcohol dehydrogenase.

Overall, we demonstrated the broad applicability of our lab-on-a-chip microfluidic spray to study biomolecules across a wide range of biological scales, from a few nanometers to a few micrometers, while preserving their morphology and heterogeneity. The spray deposition facilitated the single-molecule analysis for a few reasons. It resulted in excellent spatial separation between molecules, such as colloids that typically cluster in bulk solution because of hydrophobic effects, and we observe minimal stain deposits; in combination, these features facilitate high-throughput imaging and analysis (*21*). Finally, as we demonstrate quantitatively in the next section (Fig. 3), through structural analysis, our approach enables us to preserve not only morphology and heterogeneity but also the molecular conformation of the samples of interest under physiological-like conditions.

### Protein mass and secondary structure determination at nanogram concentrations

Next, we applied our lab-on-a-chip microfluidic spray to vibrational spectroscopy measurements on surfaces. Vibrational spectroscopy allows the characterization of chemical and structural properties of biomolecules. FTIR-ATR provides high sensitivity levels at relatively low concentrations of 1 to 10 mg/ml in air and 10 to 100 mg/ml in liquid (*2*). However, the higher concentrations needed in liquid can destabilize the biomolecules and often do not reflect their properties at physiological concentrations, while the drying process to measure in air has substantial effects on the sample and spectral quality. In the case of protein solution containing stabilizing buffer salts, salt crystallization during drying can perturb the IR spectrum due to absorption and scattering effects (fig. S6) (*22*). To prevent these issues, reduction of the droplet size was advocated in previous studies to shorten drying times. Efforts have been made in this context to generate nanoliter to picoliter droplets by means of spraying systems such as pneumatic and ultrasonic nebulizers, thermospray, and electrospray (*23*–*29*). These efforts have resulted in spectra acquired at protein quantities as low as 1 μg; however, this is in nonbiological solvents or in pure water.

**Fig. 4.**
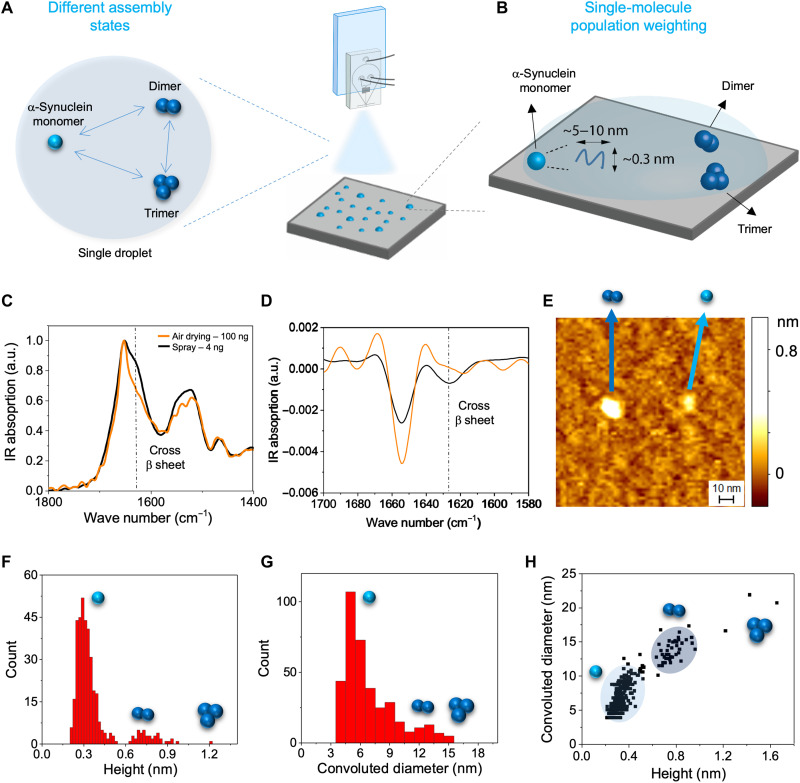
Conformational properties of the early assemblies of in an amyloid formation process. (**A**) Single-step spray deposition enables the quantitative investigation of the different self-assembled states of α-synuclein using single-molecule AFM. (**B**) Ultrafast drying better maintains the conformation of monomeric α-synuclein, compared to conventional preparation methods, as determined by ATR-FTIR. Thus, sample heterogeneity and conformations of the protein in solution can be represented on a 2D surface and imaged using AFM. (**C** and **D**) Comparison of the ATR-FTIR spectra of α-synuclein deposited via conventional air drying and via microfluidic spray deposition. A shoulder is observed at 1625 cm^−1^ for the manually deposited samples, indicating the presence of the characteristic cross β structure of amyloid fibrils, which is not observed for the sprayed sample. This is further demonstrated by the second derivative analysis of the amide I band in (**D**). (**E** to **H**) Samples were deposited via microfluidic spray directly onto mica and imaged via AFM. Monomers, dimers, and trimers can be resolved. The cross-sectional height (**F**) and diameter (convoluted by tip effects) (**G**) were measured for all species. From these measurements, the populations of monomers, dimers, and trimers were determined (**H**).

To demonstrate the capabilities of our device to acquire IR spectra under near-physiological salt conditions in a dry state, we characterized the structure and molecular weight of both globular (Fig. 3) and intrinsically disordered (Fig. 4 and fig. S9) proteins with different secondary structural contents. We compared the conformations of protein deposited via manual deposition in liquid (Fig. 3A), via dried (Fig. 3B), and via microfluidic spray (Fig. 3C).

First, these measurements enabled the assessment of the sensitivity of our spray approach compared to manual deposition in air and in liquid, as illustrated in the case of thyroglobulin (Fig. 3, D to F). Spectra were reliably acquired in liquid (Fig. 3D) down to 12 μg of protein, but lower sample concentration resulted in distortions of spectra (note S6). Thus, to measure lower concentrations, FTIR spectra were acquired in air (dried samples) (Fig. 3E). However, the presence of buffer salts resulted in spectral distortion at a sample mass of 1 μg. Reliable spectra were acquired after a rinsing step to remove salts, which allowed us to measure spectra using 100 ng of protein. However, the rinsing step can perturb the heterogeneity and molecular conformation of the sample. Our single-step spray deposition enabled the characterization of extremely small amounts of protein on the surface of the IR prism in the presence of salt (Fig. 3G), as low as 3 to 4 ng, while fully preserving spectral quality for structural and chemometric characterization (Fig. 3, F and G). We also measured the signal-to-noise ratio (SNR) of spectra at each concentration (Fig. 3G and Materials and Methods), demonstrating a 1000-fold increase in sensitivity when using microfluidic spray deposition compared to measurements in liquid and 100-fold compared to traditional slow drying and rinsing. This is comparable with the latest advances in vibrational spectroscopies equipment and preparation methods (*23*–*25*, *30*). The improvement in sensitivity that we achieved can be rationalized by considering the reduced salt crystallization occurring with fast-drying microdroplets (fig. S6 and note S7).

We next sought to show that our lab-on-a-chip microfluidic spray deposition preserves the structural state of the sample during drying, similarly to when studied in a hydrated state in liquid. We compared the spectra of several globular proteins, measured in liquid at high concentrations and when deposited by spray (Fig. 3H, left, and figs. S6 to S8). The spray spectra presented only minor differences with the secondary structure of protein in liquid (<10%), as shown by the second derivative analysis of the amide I band (Fig. 3H, right). The use of globular proteins with different secondary structural components also ensures that we have broad applicability to various protein folds (fig. S7).

Finally, we demonstrated simultaneous molecular mass and structure determination of protein with high accuracy (Fig. 3I and Materials and Methods). We can control the amount of protein deposited onto the surface of the prism with high levels of reproducibility by controlling the spray parameters. The area of the amide I band is directly proportional to the number of amino acids present on the surface (Fig. 3I, left). Thus, the area of this band will be directly proportional to the mass of protein sprayed on the prism, which can be calculated knowing the molecular weight of the protein used, the flow rate, the spray time, and the percentage of protein that is sprayed directly on the sensitive area of the FTIR-ATR prism (fig. S3). In Fig. 3I (middle), we show the measured mass using a solution of thyroglobulin protein to obtain a calibration curve (black linear fit). Then, by measuring the area of the amide I band of an unknown protein, we back-calculated its mass and molecular weight (see Materials and Methods), thus allowing simultaneous identification of protein secondary structure and molecular weight (Fig. 3I, right).

Overall, our microfluidic spray deposition enables us to overcome the current limitations of conventional IR and vibrational methods. We demonstrate the ability to perform simultaneous chemometric mass and secondary structure determination of unknown proteins down to the order of a single nanogram under physiological-like conditions, thus mimicking protein structure and concentrations in vivo (*23*–*28*, *30*).

### Conformational properties of the early assemblies of an amyloid formation process

Above, using the lab-on-a-chip microfluidic spray deposition with ultrafast drying, we demonstrated that it is possible to preserve both sample heterogeneity and conformation, enabling the quantitative study of protein aggregates using EM and vibrational IR spectroscopy. In this section, we exploit these benefits of the microfluidic spray to generate new information about a notoriously difficult to study amyloidogenic protein sample containing heterogeneous, dynamic assembly states.

Traditional preparation methods may frustrate the quantitative study of heterogeneous species present in the self-assembly of amyloidogenic proteins, because of selective adsorption effects and surface-induced self-assembly. In this section, we demonstrate that the capabilities of our method can be applied to quantitatively study the conformational heterogeneity of a freshly filtered solution of α-synuclein protein during its early molecular assembly, which is directly involved in the onset of Parkinson’s disease and neurodegeneration (Fig. 4, A and B).

We first used vibrational ATR-FTIR spectroscopy to acquire information about the conformational state of the aggregation-prone solution of α-synuclein and compared results from manual or spray deposition (Fig. 4C). To assess protein secondary structure, we studied the shape of the amide I band by second derivative analysis (*31*). The sample deposited by traditional drying methods showed two major peaks (Fig. 4D): (i) The first is related to α-helical conformation at 1654 cm^−1^; (ii) the second is related to intermolecular β-sheet content at 1625 cm^−1^, thus demonstrating that unwanted surface-driven aggregation into amyloid species is occurring on the ATR prism. The IR spectrum of α-synuclein deposited by microfluidic spray exhibited instead only a single major peak at 1645 to 1654 cm^−1^, indicating the presence of random coil and α-helical conformations, which are related to the presence of monomeric and oligomeric species. Therefore, compared to manual deposition, the microfluidic spray deposition maintained the conformation of the solution of α-synuclein closest to its conditions in the bulk.

Having demonstrated the preservation of the molecular conformation of α-synuclein as in solution, we combined our microfluidic spray deposition with high-resolution AFM (Fig. 4E) to identify single-assembly states present. The high IR sensitivity afforded to us by the spray deposition allowed us to conduct our FTIR and AFM experiments at the same protein concentration, thus facilitating direct comparison between chemical and single-molecule structural characterization. A fresh preparation of α-synuclein was deposited directly onto mica using microfluidic spray. Crucially, fast drying enables the characterization of transient assembly states, which would otherwise not be detected because of the long incubation times associated with traditional deposition. We exploited this to accurately identify the heterogeneous and transient populations during the early assembly of α-synuclein.

AFM is capable of measuring the morphology of the individual assemblies at the earliest time points of aggregation, down to nanometer resolution (Fig. 4, F and G), allowing us to distinguish between single monomers, dimers, trimers, and larger oligomers (*32*, *33*). We measured the cross-sectional height and diameter of each assembly state on the surface and identified the species present as being monomers, dimers, and trimers based on their height, which were around 0.3, 0.6, and 1.2 nm, respectively, as we previously demonstrated (Fig. 4G) (*3*, *33*). Few oligomers with larger cross-sectional dimensions were observed. Then, we counted the different species per unit of surface and determined the relative abundance of each species in the first minutes of the early molecular assembly. As we deposited biomolecules on the surface in a single step with no rinsing, we assumed that we maintain the full heterogeneity of our sample. Thus, the relative weighting of the populations on the mica surface correspond to those in solution. We determined a ratio between monomers, dimers, and trimers of about 90:8:2%. This capability is key because early oligomeric species are currently considered the putative species for the disease onset and the knowledge of exactly what and how much of each species is present will enable us to better understand this complex aggregation process (*34*).

## DISCUSSION

We have presented a general lab-on-a-chip spray for the analysis of biomolecular systems on surface by various bulk and nanoanalytical methods (Fig. 1). The lab-on-a-chip mixing capabilities allow us to stain and dilute samples for a wide range of single-molecule techniques (Fig. 2), while the standardized setup resulted in fine control over sample deposition with a high level of reproducibility. Next, we applied this setup to achieve high-sensitivity chemical identification and structural characterization of proteins in physiological-like buffers, with equivalent levels of sensitivity as measurements acquired with state-of-the-art methodology under nonphysiological conditions, such as in pure water (Fig. 3) (*23*–*25*, *27*, *28*, *30*).

Overall, we have demonstrated that this approach preserves the heterogeneity and molecular conformation of transient biomolecules over multiple biological scales and in salt-containing solution, thereby more faithfully representing the physiological state of biomolecules. The ability to prepare samples for surface-based techniques in ways that maintain conformations and assemblies present in solution, while minimizing the influence of salt, opens up a fruitful avenue to study biological systems that were previously inaccessible to characterization on surfaces. We have also demonstrated the value of these capabilities to study the morphological, structural, and population weighting of the transient and nanoscale-sized oligomeric populations formed by α-synuclein during its self-assembly (Fig. 4). The study of these populations is of fundamental importance to elucidate the molecular basis of the onset of neurodegeneration.

Last, the advances in bulk and nanoanalytical chemistry capabilities presented here create the basis for future work, extending the experimental possibilities of surface-based methods to include notoriously difficult to study systems, such as those that are sensitive to environmental changes or that exist in low abundance. We also anticipate the possible applications to other analytical techniques, such as Raman spectroscopy or cryo-EM. In the case of cryo-EM, the fast droplet drying minimizes ice crystallization and allows for quantitative experiments with a high level of statistical power. The extensive characterization of the behavior of both solvent and biomolecules when sprayed (*1*, *12*) enables fine control over relevant processes such as droplet evaporation and film thickness, which currently represent a major challenge (*21*, *35*, *36*). Furthermore, the lab-on-a-chip capabilities, which shorten experimental time scales from minutes to seconds, combined with ultrafast drying, can enable time course studies of fast-reacting systems, which represent an attractive experimental approach. While we have only presented lab-on-a-chip capabilities in the context of a double-inlet, mixing device, we also note the possibility for facile methodological extension to further lab-on-a-chip capabilities, such as on-chip reactions and desalting. In addition, the ultrafast drying essentially acts as a “freeze-frame,” opening the door to characterize transient phenomena in a time-dependent manner. These experimental capabilities offer the opportunity to efficiently characterize biomolecules while minimizing sample use, which is particularly beneficial for precious samples that require complex preparation or are obtained from human biopsies.

## MATERIALS AND METHODS

### Production of the Aβ_40_ and Aβ_42_ peptides

Expression and purification of the recombinant Aβ (M1-40) peptide (MDAEFRHDSGYEVHHQKLVFFAEDVGSNKGAIIGLMVGGVV), denoted Aβ40, and Aβ (M1-42) peptide (MDAEFRHDSGYEVHHQKLVFFAEDVGSNKGAIIGLMVGGVVIA), denoted Aβ42, were carried out as previously described (*17*). Aβ40/Aβ42 was expressed in the *E. coli* BL21 Gold (DE3) strain (StrataGene, La Jolla, CA) and purified by sonication and dissolving the inclusion bodies in 8 M urea, followed by ion exchange in batch mode on diethylaminoethyl cellulose resin. Fractions containing Aβ40/Aβ42 were lyophilized and further purified using a Superdex 75 HR 26/60 column (GE Healthcare, Chicago, IL), and eluates were analyzed using SDS–polyacrylamide gel electrophoresis for the presence of the desired protein product. The fractions containing the recombinant peptides were combined, frozen using liquid nitrogen, and lyophilized either in 20 mM sodium phosphate buffer (pH 8) and 0.2 mM EDTA (for Aβ42) or in 50 mM ammonium acetate (pH 8.5) (for Aβ40). The lyophilized product was then stored at −80°C.

### Production of α-synuclein

Recombinant α-synuclein was expressed into BL21-competent cells and purified as described previously with slight modifications . Cell pellets were resuspended in 10 mM tris (pH 8.0), 1 mM EDTA, and 1 mM phenylmethylsulfonyl fluoride and lysed by multiple freeze-thaw cycles and sonication. The cell suspension was boiled for 20 min and centrifuged at 13,500 rpm with a JA-20 rotor (Beckman Coulter, Brea, CA). Streptomycin sulfate was added to the supernatant to a final concentration of 10 mg/ml, and the mixture was stirred for 15 min at 4°C. After centrifugation at 13,500 rpm, the supernatant was taken with an addition of ammonium sulfate (0.36 g/ml). The solution was stirred for 30 min at 4°C and centrifuged again at 13,500 rpm. The pellet was resuspended and dialyzed overnight against buffer containing 25 mM tris (pH 7.7) and 1 mM EDTA. Ion exchange chromatography was then performed using a Q Sepharose HP HiScale 26/20 of buffer A [25 mM tris (pH 7.7) and 1 mM EDTA] and buffer B [25 mM tris (pH 7.7), 1 mM EDTA, and 1 M NaCl]. The fractions containing α-synuclein were pooled together and further purified using a HiLoad 26/600 Superdex 75-pg column (GE Healthcare, Chicago, IL) in 20 mM NaP (pH 6.5) and 1 mM EDTA. α-Synuclein samples were lastly pooled and stored as frozen aliquots (−20°C).

### Preparation of Aβ42 fibrils

Solutions of the lyophilized Aβ42 monomers were first dissolved in 6 M guanidinium hydrochloride and purified from any other oligomeric species and salts using a Superdex 75 10/300 GL column (GE Healthcare, Chicago, IL) at a flow rate of 0.5 ml/min in 20 mM sodium phosphate buffer (pH 8) and 0.2 mM EDTA. The center of the peak corresponding to the monomeric protein was obtained, and the concentration was determined by absorbance using the extinction coefficient ε_280_ = 1490 M^−1^ cm^−1^. The obtained monomer was diluted with buffer to 5 μM and pipetted into multiple wells of a 96-well, half-area, black/clear flat-bottom polystyrene NBS (non-binding surface) plate (3881, Corning, NY) at 100 μl per well. Samples were then incubated at 37°C in a plate reader (FLUOstar Optima, BMG Labtech, Ortenberg, Germany). The fibrillation process was followed by monitoring the fluorescence over time for additional Aβ42 samples that were also supplemented with 20 μM thioflavin-T (ThT). Once the fibrillation plateau was observed via fluorescence, fibrillar samples without ThT were collected from the wells into low-binding tubes.

### Preparation of Aβ40 oligomers

Aβ oligomers were prepared as previously described with slight modifications (*37*). The lyophilized Aβ40 peptide (0.5 mg) was dissolved in 300 μl of 100% hexafluoroisopropanol (HFIP) overnight at 4°C. The solvent was then evaporated under a gentle stream of N_2_. Dimethyl sulfoxide (DMSO) was then added to yield a peptide concentration of 2.2 mM, which was then sonicated twice at room temperature using a bath sonicator. The peptide was finally diluted in 20 mM sodium phosphate buffer (pH 6.9) with 200 μM ZnCl_2_ to a final concentration of 100 μM. This solution was incubated at 20°C for 20 hours, after which the oligomers were collected by centrifuging the sample at 15,000*g* for 15 min at 25°C and resuspending the pellet in 20 mM sodium phosphate buffer (pH 6.9) with 200 μM ZnCl_2_.

### Preparation of small-molecule colloids

Bexarotene was obtained from Sigma-Aldrich (St. Louis, MO) at the highest purity available. Colloid formation was performed by first dissolving the bexarotene power in 10 mM solution stocks of 100% DMSO, before a serial dilution in 20 mM NaP (pH 8.0) and 0.2 mM EDTA to 100 μM in 1% (v/v) DMSO (*38*).

### Preparation of large unilamellar vesicles

1-Palmitoyl-2-oleoyl-*sn*-glycero-3-phospho-l-serine (POPS) and 1-palmitoyl-2-oleoyl-*sn*-glycero-3-phosphocholine (POPC) were purchased from Avanti Polar Lipids Inc (Alabaster, AL). POPC and POPS in chloroform solutions were first mixed at a 7:3 lipid equivalent ratio. The solvent was then evaporated using a gentle stream of N_2_. Subsequently, POPC:POPS lipid films were dissolved in 20 mM NaP (pH 8.0) to a stock concentration of 2 mM and stirred at 45°C for 2 hours. The solution was subjected to 5 cycles of freeze-thaw, after which the preparation of large unilamellar vesicles was done via extrusion through a 200-nm pore diameter membrane (Avanti Polar Lipids Inc.)

### Preparation of *E. coli*

Glycerol stocks of previously transformed *E. coli* cells with the above recombinant Aβ42 sequence and an ampicillin resistance gene were used to inoculate overnight cultures in sterile conical flasks containing LB medium with ampicillin (100 μg/ml). Solutions were harvested the next day to collect the *E. coli* samples.

### Preparation of globular proteins

For FTIR measurements, proteins were dissolved in 25 and 50 mM tris-HCl and 50 and 100 mM KCl (pH 7.4) to a concentration of 8 mg/ml. They were then filtered through a 22 μM pore and centrifuged at 22,000*g* for 3 min at 4°C and then resuspended in fresh buffer and stored at −20°C until use. Proteins were then diluted in the same buffer to the desired concentration immediately before the experiment. All proteins were acquired from Sigma-Aldrich.

### Fabrication of microfluidic spray devices

A two-step photolithographic process was used to fabricate the master used for casting microfluidic spray devices. Briefly, a 25-μm-thick structure was fabricated (3025, MicroChem) was spin-coated onto a silicon wafer. This was then soft-baked for 15 min at 95°C. An appropriate mask was placed onto the wafer, exposed under ultraviolet light to induce polymerization and then post-baked at 95°C. A second 50-μm-thick layer (SU-8 3050, MicroChem) was then spin-coated onto the wafer and soft-baked for 15 min at 95°C. A second mask was then aligned with respect to the structures formed from the first mask, and the same procedure was followed, i.e., exposure to ultraviolet light and post-baking for 15 min at 95°C. Last, the master was developed in propylene glycol methyl ether acetate (Sigma-Aldrich) to remove any photoresist that had not cross-linked.

A 1:10 ratio of polydimethylsiloxane (PDMS) curing agent to elastomer (SYLGARD 184, Dow Corning, Midland, MI) was used to fabricate microfluidic devices. The mixture was cured for 3 hours at 65°C. The hardened PDMS was cut and peeled off the master. The two complementary PDMS chips are then activated with O_2_ plasma (Diener Electronic, Ebhausen, Germany) and put in contact with each other and aligned precisely such that the gas inlet intersects with the liquid inlet to form a 3D nozzle (*12*).

### Use of microfluidic spray devices

Before introduction of the sample, each device was tested and washed with MilliQ water for 5 min. The sample was then loaded into either 1-ml air-tight plastic syringes (norm-Ject) or 200-μl air-tight glass syringes (Hamilton, Reno, NV) and driven into the spray device using a syringe pump (Harvard Apparatus, Hollisten, MA). Solutions containing sample, or 2% (w/v) uranyl acetate for EM experiments, were pumped into the device with a flow rate of 100 μl/hour, while the nitrogen gas inlet pressure was maintained at 3 bar. Deposition was conducted for a maximum of 10 s at a distance of 3.5 cm to ensure that coalescence did not occur. Samples were sprayed directly onto the relevant surfaces (i.e., mica, FTIR prism, or TEM grids) with no further washing steps required before measurements.

### Electron microscopy

Samples for TEM were prepared on 400-mesh, 3-mm copper grid carbon support film (EM Resolutions Ltd., Sheffield, UK) and stained with 2% (w/v) uranyl acetate. The samples were imaged on an FEI Tecnai G2 transmission electron microscope (Cambridge Advanced Imaging Centre). Images were analyzed using the SIS MegaView II Image Capture system (Olympus, Tokyo, JP).

Samples for scanning EM (SEM)/scanning TEM (STEM) were prepared using the same protocol as those for TEM. The samples were imaged on a Thermo Fisher Scientific (FEI) Talos F200X G2 TEM (Waltham, MA), operated at 30 and 5 kV for STEM and SEM, respectively.

### Dynamic light scattering

All DLS experiments were performed with a Zetasizer Nano-S (Malvern Panalytical, Malvern, UK) at 25°C. The hydrodynamic radii were deduced from the translational diffusion coefficients using the Stokes-Einstein equation. Diffusion coefficients were inferred from the analysis of the decay of the scattered intensity autocorrelation function. All calculations were performed using the software provided by the manufacturer.

### FTIR spectroscopy

Measurements were performed on a Vertex 70 FTIR spectrometer (Bruker, Billerica, MA). Each spectrum was acquired with a scanner velocity of 20 kHz over 4000 to 400 cm^−1^ at an average of 128 to 412 scans. Spectra acquired in liquid were performed using a bioATR cell II unit and a mercury cadmium telluride detector. Spectra acquired in air were performed using a DiamondATR unit and a deuterated lanthanum α-alanine–doped triglycine sulfate detector. For traditional sample deposition methods, 15 μl of sample was deposited to cover the prism. For measurements taken in liquid, spectra were acquired immediately after deposition onto the sample cell. For measurements taken in air, the sample was allowed to adsorb for 5 min and then subsequently blotted and rinsed with 10 μl of MilliQ water. New background spectra were acquired before each measurement. All spectra were analyzed using OPUS software (Bruker) and OriginPro (Origin Labs, Northampton, MA). Spectra presented represent three individual spectra that were averaged and normalized. To determine the secondary structure composition of proteins, a second derivative analysis was performed. Spectra were first smoothed by applying a Savitzky-Golay filter. To assess the sensitivity of each deposition method, i.e., spray, liquid, and air, the SNR were calculated, both for our own data and for other innovative sample deposition methods reported in the literature. The SNR was determined by dividing the intensity of the amide I peak by the root mean square of the instrument noise. The SNR was then plotted versus the mass of protein deposited onto the prism (discussed below). A linear fit was applied to extrapolate the minimum mass required to achieve a spectrum, which is in excellent agreement with experimental data.

### Sample deposition on FTIR cell holder calculation

To estimate the distribution of sprayed sample arriving on the cell holder, a calibration using fluorescence microscopy was first conducted. A solution (0.1 mg/ml) of a fluorescent dye, fluorescein, was sprayed onto a glass slide using the same parameters as those used in all spraying experiments, i.e., a distance of 3.5 cm with a pressure of 3 bar and a flow rate of 100 μl/hour. Fluorescent images were taken at regular spatial intervals across the glass slide (fig. S2, C to E). By calculating the fluorescence intensity acquired from each image, the distribution of the sprayed fluorescein was plotted (blue points in fig. S2B) and fitted to a Gaussian (red curve in fig. S2B). Assuming that a similar distribution is observed when protein sample is sprayed onto the FTIR cell holder, we estimated the amount of protein that actually lands on the FTIR prism. As the prism has dimensions of 2 mm by 2 mm and if the spray nozzle is placed exactly above the center of the cell holder, then the amount of protein sample sprayed onto the holder is represented by the pink area in fig. S2B. Dividing this area by the total area under the distribution, we find that 8.9% of the total protein solution sprayed lands onto the FTIR prism.

### Protein molecular weight estimation from FTIR spectra

Protein samples were sprayed onto the sample holder, and their FTIR spectra were obtained. The area of spraying is accurately determined as shown in fig. S3. Thus, by varying spray times, for instance, from 2 to 10 s, one can vary the amount of protein that reaches the prism. The area under the amide I band of the ATR-FTIR spectra depends on the amount of amino acids absorbing the IR light and therefore on the amount of protein on the ATR-FTIR prism. Because the concentration of protein used, the molecular weight and the flow rate at which the sample was sprayed is known, and by using the fact that 8.9% of the total spray reaches the ATR-FTIR prism (fig. S3), the mass deposited onto the FTIR holder could be calculated. We used thyroglobulin as a calibration protein with a known molecular weight (669 kDa) to generate a calibration curve, which can be described using a linear fit, as was expected (Fig. 3). The molecular mass of other unknown proteins was then determined using this linear relationship as calibration curve. By spraying other proteins and obtaining their FTIR spectra, the area under the amide I band was measured; by knowing the mass and volume sprayed (from the flow rate and time of sample sprayed), we could back-calculate the molecular mass of the protein under question within an error of around 11% due to the calibration curve to the mass deposited in Fig. 3G. The error on the *y* axis is derived from the area under the amide I band. It was obtained by averaging over a range of three spectra. The error on the reported mass value is a combination of three different factors and is shown to 2 SDs, namely, the flow rate at which the liquid was flown through the microfluidic device, the spray deposition time, and the concentration of the thyroglobulin solution used for the calibration curve.

### Atomic force microscopy

Protein samples were sprayed directly onto freshly cleaved mica surfaces with no further steps. The preparation was carried out at room temperature. AFM maps were acquired using an NX10 AFM (Park Systems, South Korea) operating in noncontact mode and equipped with a silicon tip (PPP-NCHR; 42 N/m) with a nominal radius of <10 nm. All images were performed at room temperature. Image flattening and statistical analysis were performed by SPIP (Image Metrology, Denmark) software. Height and diameter were measured by taking a cross section along the *Z* direction of each biomolecule.

## Supplementary Materials

### This PDF file includes:

Supplementary Text Table S1 Figs. S1 to S10 References

## Notes

View/request a protocol for this paper from *Bio-protocol*.
