# Supplementary material for: Enhanced surface nanoanalytics of transient biomolecular processes
Source: Sci Adv. 2023 Jan 13;9(2):eabq3151. doi: 10.1126/sciadv.abq3151 (PMC9839325; doi:10.1126/sciadv.abq3151)
Supplement: Supplementary file 1 — Supplementary Text Table S1 Figs. S1 to S10 References [file sciadv.abq3151_sm.pdf]

Science Advances
AAAS

Supplementary Materials for

Enhanced surface nanoanalytics of transient biomolecular processes

Alyssa Miller *et al.*

Corresponding author: Michele Vendruscolo, mv245@cam.ac.uk;
Francesco Simone Ruggeri, simone.ruggeri@wur.nl; Tuomas P. J. Knowles, tpjk2@cam.ac.uk

Sci. Adv. **9**, eabq3151 (2023)
DOI: 10.1126/sciadv.abq3151

This PDF file includes:

Supplementary Text
Table S1
Figs. S1 to S10
References

Supplementary Text

Supplementary Note 1: Manual deposition of samples on surfaces

The fixing of bio-organic and biomolecular samples on surface-based methods is often performed by manual deposition. For most analytical methods, conventional manual surface-based sample preparation involves three fundamental steps: 1) pipetting a volume of solution onto a surface and allowing it to adsorb for up to a few minutes, 2) rinsing to remove weakly adsorbed biomolecules and excess salt present in buffer, and 3) removing solvent using gentle drying. Conventionally, steps 1 and 2 are applied to measure in a liquid solution, while step 3 is necessary to measure the sample in an air or vacuum environment.

Supplementary Note 2: Optimisation of microfluidic spray experimental set-up

Every aspect of the experimental set-up was carefully considered, such as syringe size and material (**Fig. S2**). We found low volume glass syringes (250 µl) to be ideal, as this minimises sample clogging and ensures accurate volumes of liquids are flowed through the microfluidic spray device. Glass tubing proved to be incompatible with the experimental set-up due to its lack of flexibility, therefore thin polytetrafluoroethylene (PTFE) tubing was used. A standard inlet tube length of 12 cm minimised dead volumes. This allowed us to achieve a working volume for the operation of the device as low as 20 µl for each experiment, and without any minimum requirement for sample concentration.

Supplementary Note 3: Droplet evaporation & salt crystallisation

In order to understand the droplets sizes evaporation and salt crystallisation in microfluidic spray deposition, AFM images of salt crystals were analysed. The height was measured for numerous salt crystals formed, with an average height being 95 ± 65 nm. The average droplet size was measured using optical images taken using the AFM-equipped camera. Typical droplets formed were ~8-13 µm in diameter. From the droplet size, the evaporation time can be calculated (**Table S1**). This is discussed more thoroughly in ref (*1*).

	**Droplet diameter (µm)**	**Evaporation time (ms)**
Average	12.2	12.3
Q1	8.2	5.5
Median	9.7	7.9
Q2	12.9	13.8

Table S1. Determination of the evaporation time of the droplets generated by microfluidic spray. The average droplet size was measured from optical images (n=132). Note that this value is likely an overestimate, as droplets were measured using the optical camera based on the faint coffee-ring formed of salt crystals. Smaller droplets (those a few μm in diameter) will have smaller salt crystals forming that are below the detection limit of the optical camera.

According to Burton-Cabrera-Frank theory of salt crystallisation (*39*), from the crystal size and evaporation time, we can calculate the growth rate of salt crystals using the equation

$$G = \frac{dL}{dt}.$$

Note that height was used as a measure in crystal size instead of length (L). This is due to artefacts in AFM images preventing reliable measurement of the lateral dimensions of crystals. From this, we can say the growth rate of salt crystals is ~$1x10^{-5}$ μm/s, which is in agreement with literature values (*40–42*).

Next, we applied the calculated crystal growth rate to traditional deposition methods with slow drying times. Assuming a very conservative estimate of a few minutes (180 s) drying time of a large, 10 μl droplet, the resultant crystal would be at least a few μm (1.8 μm) in size.

Supplementary Note 4: In-flight droplet evaporation

In addition to the droplet drying on the surface, discussed above and in previous work, we must also consider the in-flight droplet drying. By means of a high-speed camera, we have previously measured the approximate velocity of the droplets to be approximately 20-30 ms^{-1} (*1*, *12*). From this, we can estimate the time of flight to be ~1-2 ms, based on the known distance between the nozzle and the surface (in this case, 3.5 cm). Taking the median droplet size of 9.7μm (Table S1), we can calculate the drying time on the surface to be ~8 ms. Therefore, the relative time of flight of the droplet compared to the time as a liquid on the surface is about 12-25%.

Supplementary Note 5: Comparison of size distributions measured from EM

The size distribution of samples deposited via microfluidic spray and manual deposition was compared to assess the preservation of sample heterogeneity of both methods. Comparisons were made to bulk DLS measurements acquired in solution. The size of the Aβ oligomers measured via spray was measured in the 2-20 nm range, compared to manual which displayed a 2-10 nm range.

As bulk DLS measurements are often not compatible with measuring oligomers due to the heterogeneous, large amorphous assemblies which can form, we instead opted to compare these EM measurements with single-molecule AFM studies. AFM is particularly well-suited for the accurate size characterisation of oligomers due to the high resolution of the technique and the lack of staining step. AFM analysis showed a 1-25 nm diameter range, which is in excellent agreement with spray EM results (**Fig. 2f**).

Next, we sought to assess the size distribution of colloids. An analysis was not possible for manual preparation, as the colloid aggregation upon manual deposition prevented their accurate measurement. It is expected that colloids will cluster in solution to form a few large particles, which will then be over-represented in DLS measurements; this was not an issue with our spray measurements. Despite the presence of large (>1000 nm) particles observed via DLS, an excellent correspondence was observed between spray deposition EM and bulk DLS measurements, with most colloids being between 5-25 nm in diameter (Q1-Q3 11-56 nm) (**Fig. S4**).

Lastly, we assessed the size distribution of lipid vesicles, which displayed a broader range for spray deposition compared to manual, with sizes observed between 40-600 nm (Q1-Q3 116-365 nm) and 100-400 nm (Q1-Q3 177 – 296 nm), respectively (**Fig. 2f** and **Fig. S4**).

<u>Supplementary Note 6: Sensitivity of FTIR measurements acquired in liquid & air</u>

Measurements of thyroglobulin acquired in liquid (**Fig. 3d**) resulted in reliable spectra which reflect a conformationally-stable protein down to 12 μg. However, low sample concentration resulted in significant distortions of spectra due to difficulty in compensating for the IR absorption of water. Water predominantly absorbs IR light at ~3500 cm^{-1} (symmetric and asymmetric O-H stretching) and at ~1600 cm^{-1} (O-H bending). In particular, the IR absorption of water overlaps with the amide I band of protein, which is key for structural analysis of protein.

Thus, to measure lower concentrations, FTIR-ATR spectra are typically acquired using dried samples (**Fig. 3e**). However, the drying of salt in the buffer results in significant spectral distortion, which hampers chemometrics and structural analysis. Reliable spectra can be achieved using smaller amounts of protein by the addition of an extra rinsing step to remove excess salt, which allowed us to retrieve spectra with high quality amide band I and II peaks and with a good signal to noise ratio. However, the rinsing step can perturb the heterogeneity and molecular conformation of the sample. Our single-step lab-on-a-chip spray deposition enabled the characterization of extremely small amounts of protein in the presence of salt. The ability to measure spectra of small amounts of protein in salt, and associated improvement in sensitivity that we achieved, can be rationalised by considering the reduced salt crystallisation occurring with fast drying microdroplets, when compared to the formation of large salt crystals during the drying of a macroscopic liquid droplet (**Fig. S6** and **Supplementary Note 5**).

Supplementary Note 7: Salt crystallisation effect on FTIR-ATR Spectra

In **Fig. S5**, the effect of salt is described for thyroglobulin samples prepared using microfluidic spray, and those measured in air, with and without rinsing. The difference in sensitivity of ATR-FTIR by manual and spray deposition can be attributed to the formation of salt crystals.

The absorbance, and therefore the signal intensity, of FTIR-ATR measurements is related to the penetration depth (d_p) of the evanescent wave and it is proportional because of the Beer-Lambert law to the thickness of the material deposited on the prism. Thus, the absorbance is proportional to the height (thickness) of the material on the surface a thicker film or crystal salt on the surface will produce a stronger IR absorption.

As calculated in the **Supplementary Note 3**, the slow drying (seconds to minutes) of a macroscopic droplet causes the formation of large micrometre sized salt crystals, which have significant absorption and affect the IR measurement. Based on the growth rate of salt crystals of ~1 x 10^{-5} µm/s (**Supplementary Note 3**), we estimate that the salt crystals can grow as large as a 1.8 µm in the slow drying method. Therefore, it is expected that the IR spectrum would disproportionately reflect the presence of the salt. While the fast evaporation of the sprayed droplets minimises the time for salt crystallisation to occur, thus producing smaller nanometre sized crystals that do not significantly affect the IR measurements. In fact, the average crystal size is 95.11 ± 65.38 nm in height for samples deposited by spraying (**Fig. S5**). This effect is quantified by analysing the ratio between IR absorption peaks typical of the protein and the salt in solution (**Fig. S5**). In order to quantify the relative contribution of the salt to the IR absorbance spectra, the peak at ~1050 cm^{-1} (the C-O bond in the primary alcohol groups of Tris was integrated and compared to that of the amide I peak We observed a ~2-fold increase of the ratio of the IR signal of the amide I and salt peak between microfluidic spray and air which was not rinsed, despite the concentration of protein to salt being the same in solution.

Thus, by significantly reducing the size of salt crystals, we thereby enable the acquisition of spectra in buffer with nanogram sensitivity without the need for rinsing (**Fig. S5**).

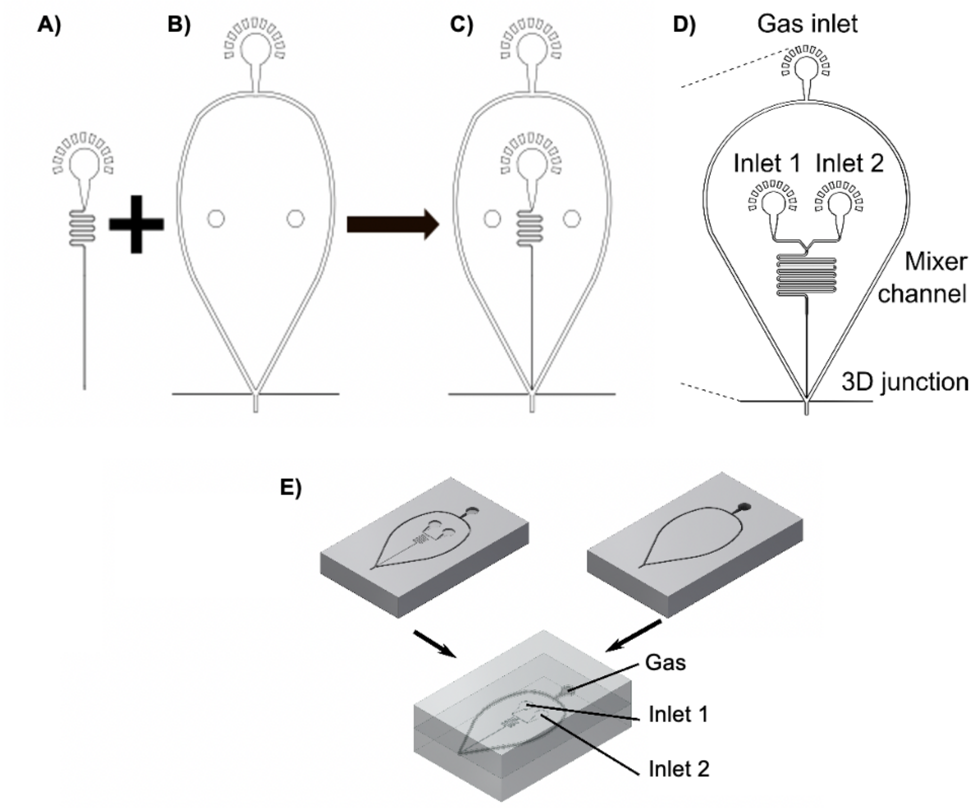

Figure S1. Schematic of the single and double inlet microfluidic device designs. (a-c) 25 μm high liquid channel (a), 50 μm high gas channel (b), and their combination using a two-step lithography process (c). The same applies for the double-inlet device, which simply has an additional liquid inlet and a longer channel to allow for sufficient mixing. (e) Assembly of the two PDMS layers occurs via plasma bonding. The PDMS pieces from the two respective masters are carefully aligned with respect to one another and left to bond to give the completed device.

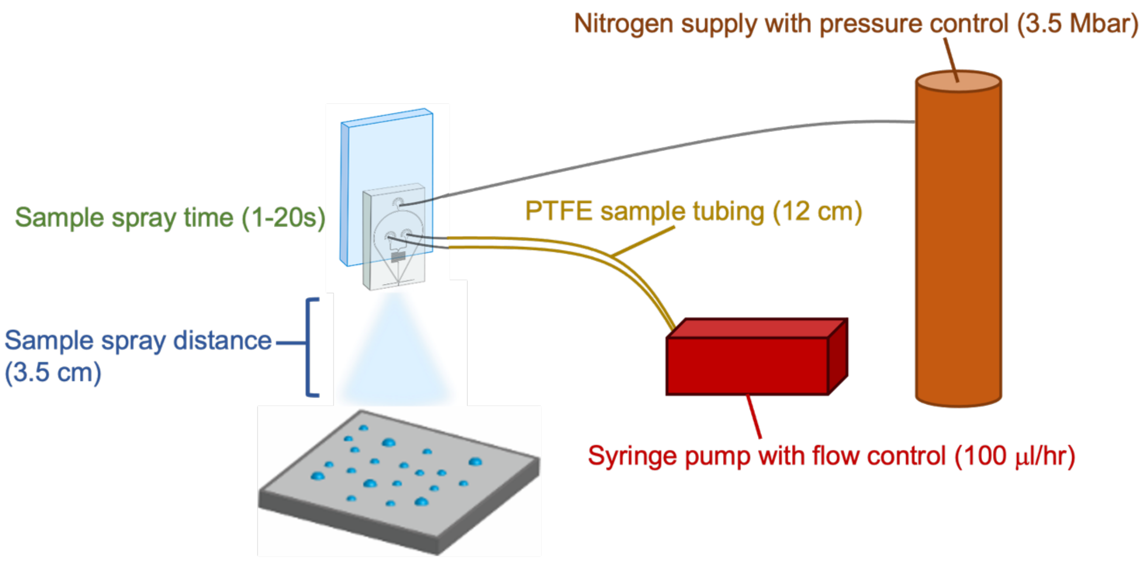

Figure S2. A cartoon depiction of the standardised experimental set-up. The following parameters were adjusted to enhance deposition reproducibility between experimentalists and to minimise sample usage: sample inlet flow rate, gas inlet pressure, tubing material and length, the time which sample is sprayed, and the distance between the spray device and the surface. A detailed description is included in the Methods section.

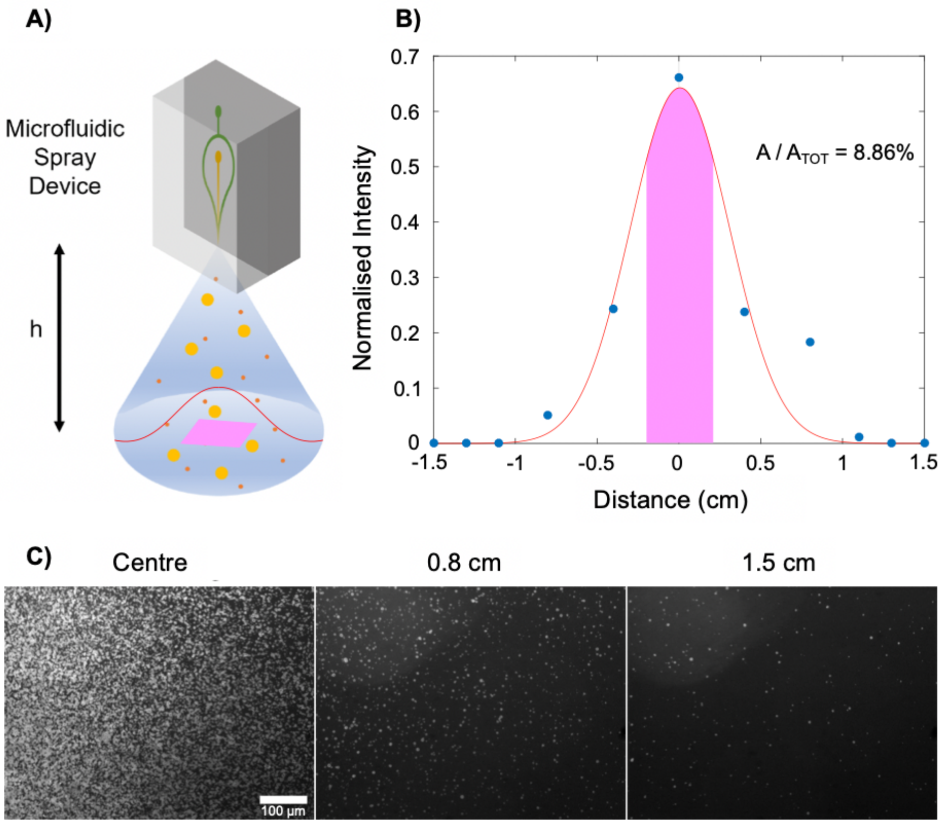

Figure S3. Characterisation of spray generated using a microfluidic device. (a) Schematic of the spray device. The spray area is represented in light blue, and the sample by the yellow and orange dots. (b) The distribution of the spray was generated by spraying fluorescein (1mg/ml) and calculating the fluorescence intensity at various positions (indicated by blue dots). The spray exhibits a Gaussian distribution (red line), with the majority of sample being deposited in the central 5 mm^2 region, as indicated by the pink area. (c) Representative fluorescence images at various distances from the spray centre show the micron-scale droplets which are generated.

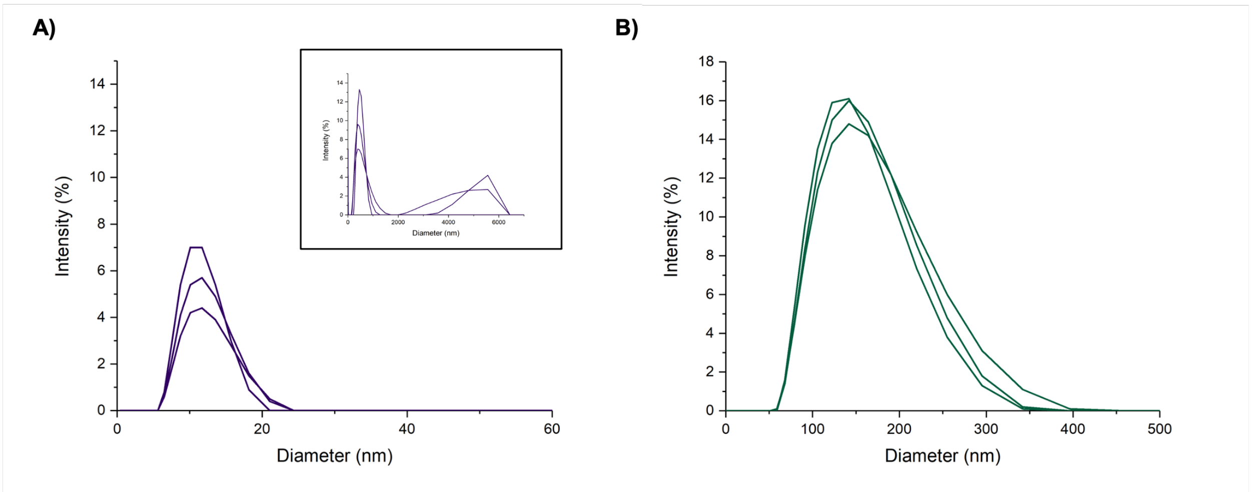

Figure S4. Solution-based measurements of particle size. Bulk DLS data showing the diameters of colloids (a) and lipid vesicles (b) in solution. We note the presence of large aggregates present in the colloid sample.

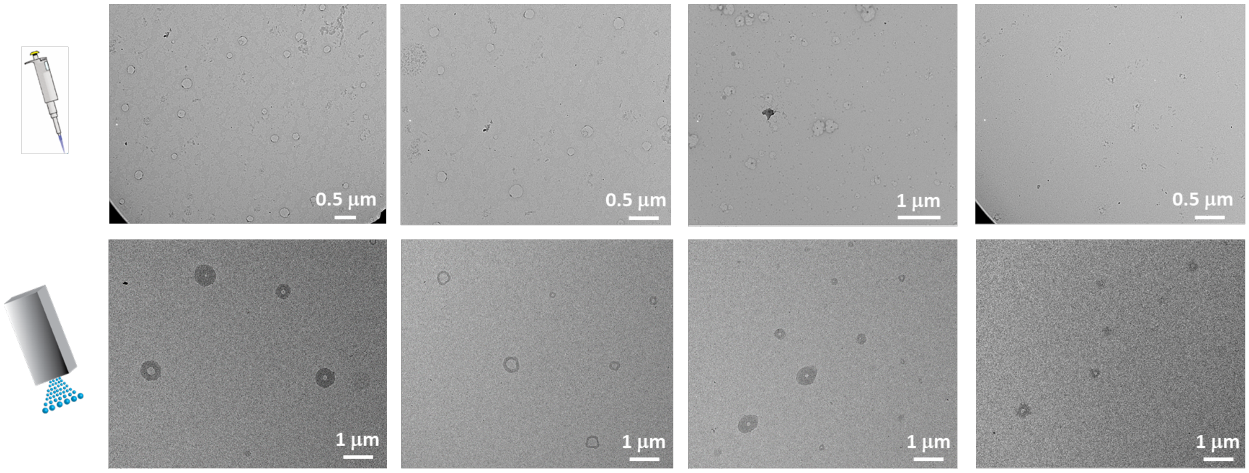

Figure S5. Spray deposition of lipid vesicles. Comparison of lipid vesicles deposited by manual (top panel) and spray (bottom panel).

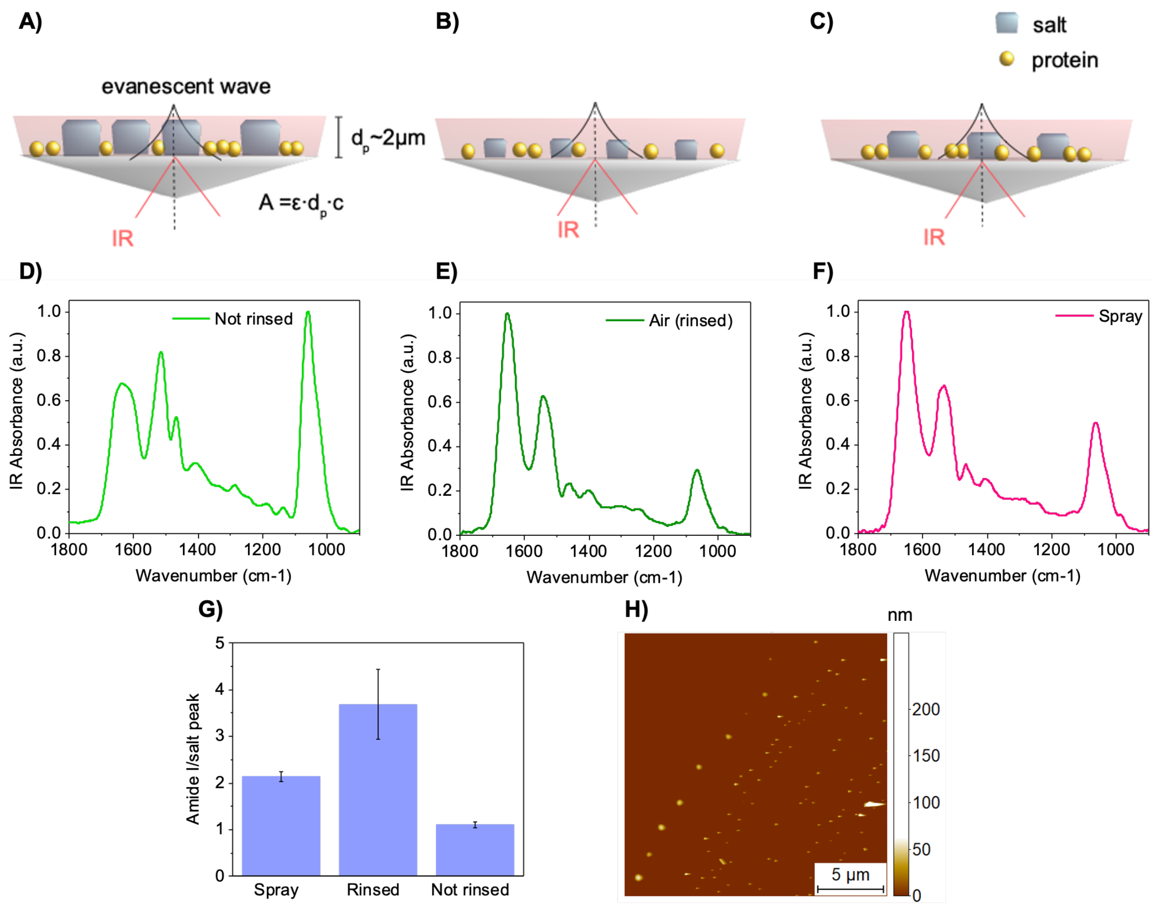

Figure S6. Influence of salt on the IR spectra. (a-f) Cartoon depicting the relative sizes of protein and salt on the FTIR-ATR prism in air: without rinsing (a), with rinsing (b) and sprayed (c) (not to scale), and the corresponding spectra of thyroglobulin (1 mg/ml) measured in buffer (d, e, f, respectively). (g) In order to understand the contributions of salt and protein to the IR spectra, the ratios between the area under the salt peak (~1050 cm^{-1}) and the amide I peak (~1650 cm^{-1}) were calculated for each preparation method. Error bars represent the SD. Despite containing the same ration between salt and protein, the relative influence of salt on the IR spectra is more important when deposited manually than when deposited via spray. (h) When samples dry slowly (as in traditional deposition methods), large salt crystals form which result in distorted protein spectra. As such, rinsing is required in order to remove salt crystals. In the case of microfluidic spray, the fast timescales of drying limit salt crystallisation and enable the easy measurement of proteins in buffer. Salt crystals were measured using AFM; a representative image is presented. A coffee-ring effect is observed, with salt crystals forming a ring-like shape at the edge of the droplet. The average crystal size is 95 ± 65 nm.

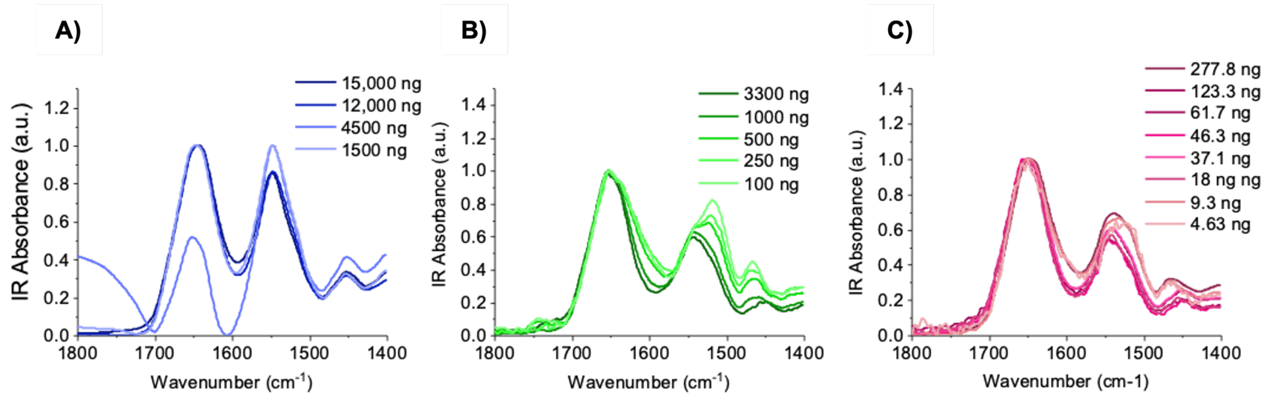

Figure S7. Thyroglobulin IR spectra at a function of mass. (a-c) Spectra are presented for thyroglobulin at a range of masses, for spectra acquired in liquid (a), air (b) and sprayed (c). Spectra are reproducible at a variety of concentrations when deposited via microfluidic spray. Where very small amounts of protein are deposited on the surface (<2 ng), the influence of salt becomes more apparent; secondary structure analysis (using second derivative) is still possible.

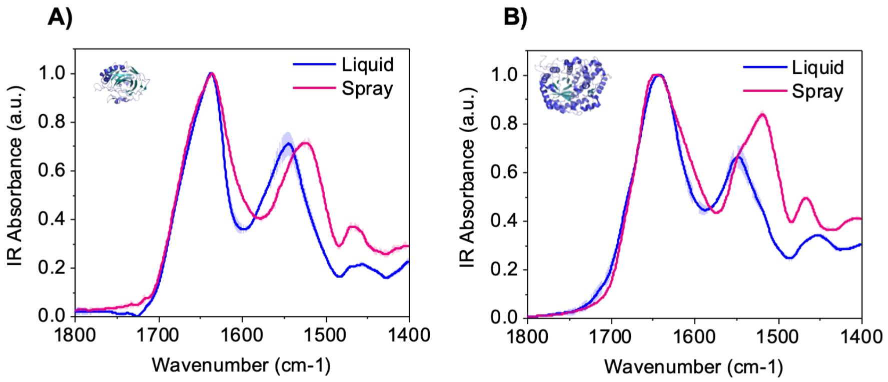

Figure S8. FTIR spectra of albumin and alcohol dehydrogenase. Spectra were obtained for samples deposited via microfluidic spray and in a liquid environment. Samples studied were albumin (a) and alchohol dehydrogenase (b).

Protein	α-helix [%]	β-sheet [%]	β-turn [%]	Coil/other [%]
α-synuclein	-	-	-	100
carbonic anhydrase (a)	16.2	31.9	27.7	24.2
β-amylase (b)	39.6	12.2	23.1	25.1
thyroglobulin (c)	21.5	15.9	30.5	32.1
albumin (d)	70.5	-	9.4	20.1
alcohol dehydrogenase (e)	25.6	29.3	15.0	30.1

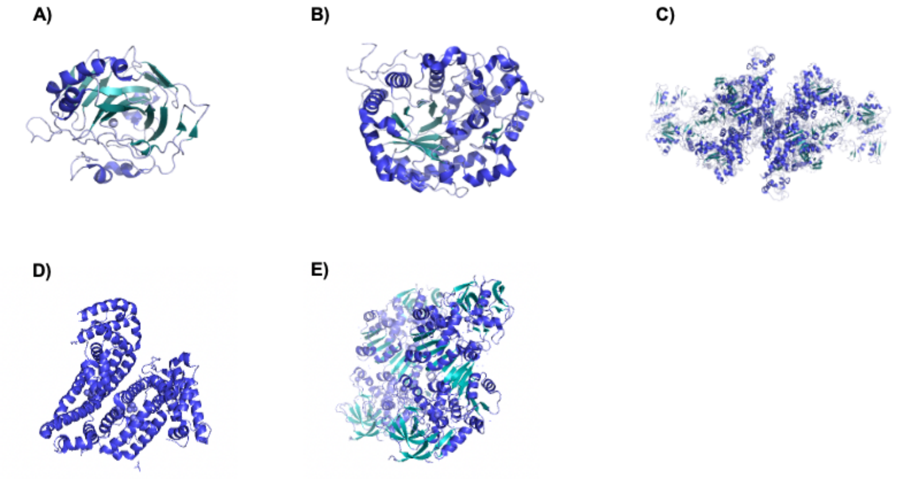

Figure S9. Properties of globular proteins. The secondary structure and corresponding structure of proteins are presented for the following proteins: carbonic anhydrase (5YUJ), β-amylase (1FA2), thyroglobulin (6SCJ), albumin (4F5T), alcohol dehydrogenase (46WZ).

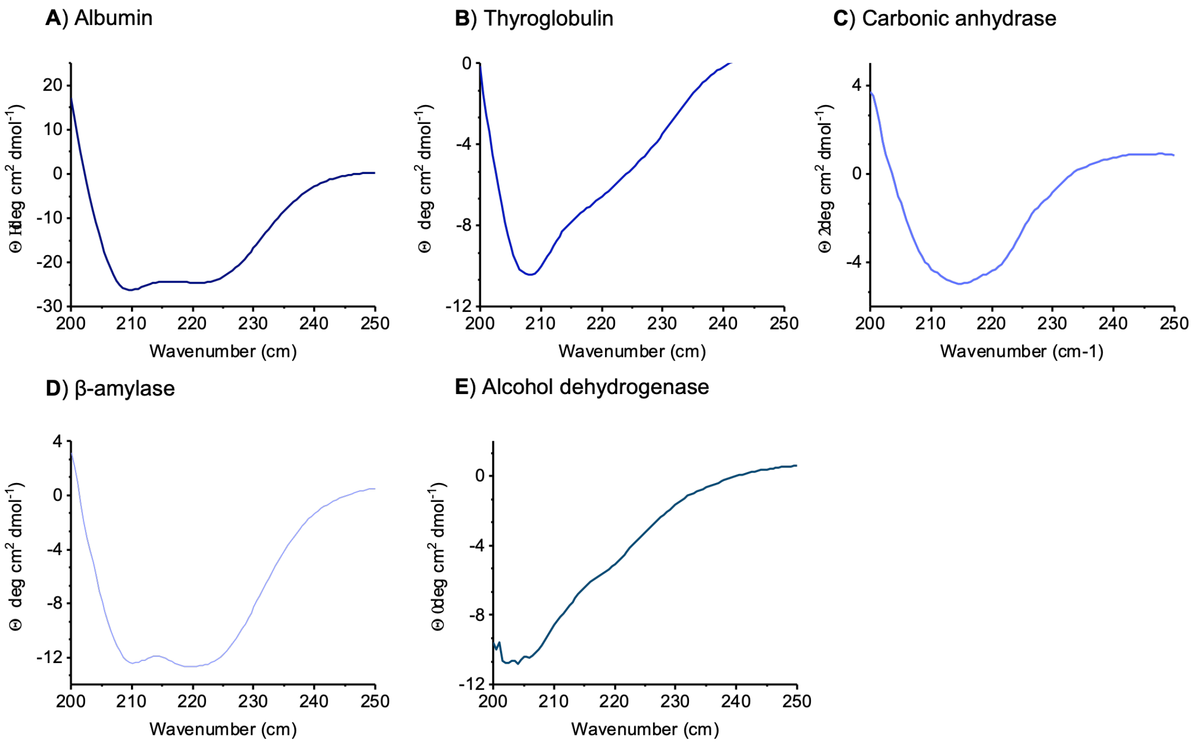

Figure S10. CD spectra of globular proteins. (a-e) CD spectra of globular proteins studied demonstrate that proteins are folded properly. Reference spectra can be found here: carbonic anhydrase (*43*), albumin (*44*), β-amylase (*45*), alcohol dehydrogenase (*46*) and thyroglobulin (*47*).

REFERENCES AND NOTES

1. F. S. Ruggeri, J. Charmet, T. Kartanas, Q. Peter, S. Chia, J. Habchi, C. M. Dobson, M. Vendruscolo, T. P. J. Knowles, Microfluidic deposition for resolving single-molecule protein architecture and heterogeneity. *Nat. Commun.* **9**, 3890 (2018).

2. E. Goormaghtigh, V. Raussens, J. M. Ruysschaert, Attenuated total reflection infrared spectroscopy of proteins and lipids in biological membranes. *Biochim. Biophys. Acta* **1422**, 105–185 (1999).

3. F. S. Ruggeri, F. Benedetti, T. P. J. Knowles, H. A. Lashuel, S. Sekatskii, G. Dietler, Identification and nanomechanical characterization of the fundamental single-strand protofilaments of amyloid α-synuclein fibrils. *Proc. Natl. Acad. Sci. U.S.A.* **115**, 7230–7235 (2018).

4. V. Chan, S. E. McKenzie, S. Surrey, P. Fortina, D. J. Graves, Effect of hydrophobicity and electrostatics on adsorption and surface diffusion of DNA oligonucleotides at liquid/solid interfaces. *J. Colloid Interface Sci.* **203**, 197–207 (1998).

5. M. Kastantin, B. B. Langdon, D. K. Schwartz, A bottom-up approach to understanding protein layer formation at solid–liquid interfaces. *Adv. Colloid Interface Sci.* **207**, 240–252 (2014).

6. D. Losic, L. L. Martin, β-Amyloid fibril formation is promoted by step edges of highly oriented pyrolytic graphite. *Biopolymers* **84**, 519–526 (2006).

7. T. Kowalewski, D. M. Holtzman, In situ atomic force microscopy study of Alzheimer's β-amyloid peptide on different substrates: New insights into mechanism of β-sheet formation. *Proc. Natl. Acad. Sci. U.S.A.* **96**, 3688–3693 (1999).

8. S. J. Prestrelski, N. Tedeschi, T. Arakawa, J. F. Carpenter, Dehydration-induced conformational transitions in proteins and their inhibition by stabilizers. *Biophys. J.* **65**, 661–671 (1993).

9. R. D. Tilton, A. P. Gast, C. R. Robertson, Surface diffusion of interacting proteins. Effect of concentration on the lateral mobility of adsorbed bovine serum albumin. *Biophys. J.* **58**, 1321–1326 (1990).

10. V. Chan, D. J. Graves, P. Fortina, S. E. McKenzie, Adsorption and surface diffusion of DNA oligonucleotides at liquid/solid interfaces. *Langmuir* **13**, 320–329 (1997).

11. S. Ravichandran, J. Talbot, Mobility of adsorbed proteins: A Brownian dynamics study. *Biophys. J.* **78**, 110–120 (2000).

12. T. Kartanas, Z. Toprakcioglu, T. A. Hakala, A. Levin, T. W. Herling, R. Daly, J. Charmet, T. P. J. Knowles, Mechanism of droplet-formation in a supersonic microfluidic spray device. *Appl. Phys. Lett.* **116**, 153702 (2020).

13. A. E. Kamholz, P. Yager, Theoretical analysis of molecular diffusion in pressure-driven laminar flow in microfluidic channels. *Biophys. J.* **80**, 155–160 (2001).

14. J. R. Harris, Transmission electron microscopy in molecular structural biology: A historical survey. *Arch. Biochem. Biophys.* **581**, 3–18 (2015).

15. G. G. Sgro, T. R. D. Costa, Cryo-EM grid preparation of membrane protein samples for single particle analysis. *Front. Mol. Biosci.* **5**, 74 (2018).

16. P. Tizro, C. Choi, N. Khanlou, Sample preparation for transmission electron microscopy, in *Biobanking: Methods and Protocols*, W. H. Yong, Ed. (Springer New York, 2019), pp. 417–424.

17. J. Habchi, P. Arosio, M. Perni, A. R. Costa, M. Yagi-Utsumi, P. Joshi, S. Chia, S. I. A. Cohen, M. B. D. Müller, S. Linse, E. A. A. Nollen, C. M. Dobson, T. P. J. Knowles, M. Vendruscolo, An anticancer drug suppresses the primary nucleation reaction that initiates the production of the toxic Aβ42 aggregates linked with Alzheimer's disease. *Sci. Adv.* **2**, e1501244 (2016).

18. A. Lesaine, D. Bonamy, C. L. Rountree, G. Gauthier, M. Impéror-Clerc, V. Lazarus, Role of particle aggregation in the structure of dried colloidal silica layers. *Soft Matter* **17**, 1589–1600 (2021).

19. S. T. Chuo, J. C. Chien, C. P. Lai, Imaging extracellular vesicles: Current and emerging methods. *J. Biomed. Sci.* **25**, 91 (2018).

20. R. J. Lobb, M. Becker, S. W. Wen, C. S. F. Wong, A. P. Wiegmans, A. Leimgruber, A. Möller, Optimized exosome isolation protocol for cell culture supernatant and human plasma. *J. Extracell. Vesicles* **4**, 27031 (2015).

21. L. F. Kourkoutis, J. M. Plitzko, W. Baumeister, Electron microscopy of biological materials at the nanometer scale. *Annu. Rev. Mater. Res.* **42**, 33–58 (2012).

22. N. Shahidzadeh-bonn, S. Rafa, D. Bonn, G. Wegdam, Salt crystallization during evaporation: Impact of interfacial properties. *Langmuir* **24**, 8599–8605 (2008).

23. V. E. Turula, J. A. de Haseth, Evaluation of particle beam fourier transform infrared spectrometry for the analysis of globular proteins: Conformation of β-lactoglobulin and lysozyme. *Appl. Spectrosc.* **48**, 1255–1264 (1994).

24. V. E. Turula, J. A. De Haseth, Particle beam LC/FT-IR spectrometry studies of biopolymer conformations in reversed-phase HPLC separations: Native globular proteins. *Anal. Chem.* **68**, 629–638 (1996).

25. K. A. Oberg, A. L. Fink, A new attenuated total reflectance fourier transform infrared spectroscopy method for the study of proteins in solution. *Anal. Biochem.* **256**, 92–106 (1998).

26. G. W. Somsen, T. Visser, Liquid chromatography/infrared spectroscopy, in *Encyclopedia of Analytical Chemistry* (Wiley, 2006), pp. 1–22.

27. G. W. Somsen, E. W. J. Hooijschuur, C. Gooijer, U. A. T. Brinkman, N. H. Velthorst, T. Visser, Coupling of reversed-phase liquid column chromatography and fourier transform

infrared spectrometry using postcolumn on-line extraction and solvent elimination. *Anal. Chem.* **68**, 746–752 (1996).

28. G. W. Somsen, I. Jagt, C. Gooijer, N. H. Velthorst, U. A. T. Brinkman, T. Visser, Identification of herbicides in river water using on-line trace enrichment combined with column liquid chromatography-Fourier-transform infrared spectrometry. *J. Chromatogr. A* **756**, 145–157 (1996).

29. J. G. Hinman, J. J. Hinman, B. E. Janicek, P. Y. Huang, K. S. Suslick, C. J. Murphy, Ultrasonic nebulization for TEM sample preparation on single-layer graphene grids. *Nano Lett.* **19**, 1938–1943 (2019).

30. B. Chon, S. Xu, Y. J. Lee, Compensation of strong water absorption in infrared spectroscopy reveals the secondary structure of proteins in dilute solutions. *Anal. Chem.* **93**, 2215–2225 (2021).

31. H. H. J. De Jongh, E. Goormaghtigh, J.-M. Ruysschaert, The different molar absorptivities of the secondary structure types in the amide I region: An attenuated total reflection infrared study on globular proteins. **242**, 95–103 (1996).

32. F. Simone Ruggeri, J. Habchi, A. Cerreta, G. Dietler, AFM-based single molecule techniques: Unraveling the amyloid pathogenic species. *Curr. Pharm. Des.* **22**, 3950–3970 (2016).

33. O. Khalaf, B. Fauvet, A. Oueslati, I. Dikiy, A.-L. Mahul-Mellier, F. S. Ruggeri, M. K. Mbefo, F. Vercruysse, G. Dietler, S.-J. Lee, D. Eliezer, H. A. Lashuel, The H50Q mutation enhances α-synuclein aggregation, secretion, and toxicity. *J. Biol. Chem.* **289**, 21856–21876 (2014).

34. M. Ingelsson, Alpha-synuclein oligomers—Neurotoxic molecules in Parkinson's disease and other lewy body disorders. *Front. Neurosci.* **10**, 408 (2016).

35. T. D. Allen, S. A. Rutherford, S. Murray, H. S. Sanderson, F. Gardiner, E. Kiseleva, M. W. Goldberg, S. P. Drummond, A protocol for isolating *Xenopus* oocyte nuclear envelope for

visualization and characterization by scanning electron microscopy (SEM) or transmission electron microscopy (TEM). *Nat. Protoc.* **2**, 1166–1172 (2007).

36. X. Feng, Z. Fu, S. Kaledhonkar, Y. Jia, B. Shah, A. Jin, Z. Liu, M. Sun, B. Chen, R. A. Grassucci, Y. Ren, H. Jiang, J. Frank, Q. Lin, A fast and effective microfluidic spraying-plunging method for high-resolution single-particle Cryo-EM. *Structure* **25**, 663–670.e3 (2017).

37. B. Mannini, J. Habchi, S. Chia, F. S. Ruggeri, M. Perni, T. P. J. Knowles, C. M. Dobson, M. Vendruscolo, Stabilization and characterization of cytotoxic $A\beta_{40}$ oligomers isolated from an aggregation reaction in the presence of zinc ions. *ACS Chem. Nerosci.* **9**, 2959–2971 (2018).

38. S. C. Owen, A. K. Doak, P. Wassam, M. S. Shoichet, B. K. Shoichet, Colloidal aggregation affects the efficacy of anticancer drugs in cell culture. *ACS Chem. Biol.* **7**, 1429–1435 (2012).

39. D. P. Woodruff, How does your crystal grow? A commentary on Burton, Cabrera and Frank (1951) ‘The growth of crystals and the equilibrium structure of their surfaces’. *Philos. Trans. R. Soc. A Math. Phys. Eng. Sci.* **373**, 20140230 (2015).

40. J. Desarnaud, H. Derluyn, J. Carmeliet, D. Bonn, N. Shahidzadeh, Metastability limit for the nucleation of NaCl crystals in confinement. *J. Phys. Chem. Lett.* **5**, 890–895 (2014).

41. N. Shahidzadeh, J. Desarnaud, Damage in porous media: Role of the kinetics of salt (re)crystallization. *EPJ Appl. Phys.* **60**, 24205 (2012).

42. J. Desarnaud, H. Derluyn, J. Carmeliet, D. Bonn, N. Shahidzadeh, Hopper growth of salt crystals. *J. Phys. Chem. Lett.* **9**, 2961–2966 (2018).

43. K. Borén, P. O. Freskgård, U. Carlsson, A comparative CD study of carbonic anhydrase isoenzymes with different number of tryptophans: Impact on calculation of secondary structure content. *Protein Sci.* **5**, 2479–2484 (1996).

44. M. Kluz, H. Nieznańska, R. Dec, I. Dzięcielewski, B. Niżyński, G. Ścibisz, W. Puławski, G. Staszczak, E. Klein, J. Smalc-Koziorowska, W. Dzwolak, Revisiting the conformational state

of albumin conjugated to gold nanoclusters: A self-assembly pathway to giant superstructures unraveled. *PLOS ONE* **14**, e0218975 (2019).

45. J.-C. Luo, S.-C. Wang, W.-B. Jian, C.-H. Chen, J.-L. Tang, C.-I. Lee, Formation of amyloid fibrils from β-amylase. *FEBS Lett.* **586**, 680–685 (2012).

46. M. Marolt, S. Lüdeke, Studying NAD(P)H cofactor-binding to alcohol dehydrogenases through global analysis of circular dichroism spectra. *Phys. Chem. Chem. Phys.* **21**, 1671–1681 (2019).

47. L. Pellizzari, G. Tell, G. Damante, Co-operation between the PAI and RED subdomains of Pax-8 in the interaction with the thyroglobulin promoter. *Biochem. J.* **337**, 253–262 (1999).
